# A Solution to Antifolate Resistance in Group B *Streptococcus*: Untargeted Metabolomics Identifies Human Milk Oligosaccharide-Induced Perturbations That Result in Potentiation of Trimethoprim

**DOI:** 10.1128/mBio.00076-20

**Published:** 2020-03-17

**Authors:** Schuyler A. Chambers, Rebecca E. Moore, Kelly M. Craft, Harrison C. Thomas, Rishub Das, Shannon D. Manning, Simona G. Codreanu, Stacy D. Sherrod, David M. Aronoff, John A. McLean, Jennifer A. Gaddy, Steven D. Townsend

**Affiliations:** a Department of Chemistry, Vanderbilt University, Nashville, Tennessee, USA; b Department of Medicine, Vanderbilt University Medical Center, Nashville, Tennessee, USA; c Center for Innovative Technology, Nashville, Tennessee, USA; d Department of Veterans Affairs, Tennessee Valley Healthcare Systems, Nashville, Tennessee, USA; e Department of Microbiology and Molecular Genetics, Michigan State University, East Lansing, Michigan, USA; f Department of Pathology, Microbiology, and Immunology, Vanderbilt University Medical Center, Nashville, Tennessee, USA; University of Oklahoma Health Sciences Center

**Keywords:** group B *Streptococcus*, human milk oligosaccharides, resistance, adjuvants, antifolate drugs

## Abstract

Adjuvants can be used to potentiate the function of antibiotics whose efficacy has been reduced by acquired or intrinsic resistance. In the present study, we discovered that human milk oligosaccharides (HMOs) sensitize strains of group B Streptococcus (GBS) to trimethoprim (TMP), an antibiotic to which GBS is intrinsically resistant. Reductions in the MIC of TMP reached as high as 512-fold across a diverse panel of isolates. To better understand HMOs’ mechanism of action, we characterized the metabolic response of GBS to HMO treatment using ultrahigh-performance liquid chromatography–high-resolution tandem mass spectrometry (UPLC-HRMS/MS) analysis. These data showed that when challenged by HMOs, GBS undergoes significant perturbations in metabolic pathways related to the biosynthesis and incorporation of macromolecules involved in membrane construction. This study represents reports the metabolic characterization of a cell that is perturbed by HMOs.

## Abstract (precis)

Group B *Streptococcus* is an important human pathogen that causes serious infections during pregnancy which can lead to chorioamnionitis, funisitis, premature rupture of gestational membranes, preterm birth, neonatal sepsis, and death. GBS is evolving antimicrobial resistance mechanisms, and the work presented in this paper provides evidence that prebiotics such as human milk oligosaccharides can act as adjuvants to restore the utility of antibiotics.

## INTRODUCTION

The development of antibiotics is arguably one of the most important advances in modern medicine. Antibiotics can be organized according to the cellular component or system they engage and whether they inhibit cell growth (bacteriostatic) or induce cell death (bactericidal). Although antibiotics that target cellular viability are effective, these agents impose selective pressures that foster the evolution of resistant phenotypes (1). Combination therapy has emerged as a powerful solution to resistance issues that plague monotherapy (2). This approach, which involves codosing an antibiotic with an adjuvant that potentiates its function or a second antibiotic with a different target, can improve efficacy and suppress resistance evolution (2–7).

One bacterial pathogen group that showcases multidrug resistance is group B Streptococcus (GBS) (8). GBS is a leading cause of neonatal sepsis, pneumonia, and meningitis (9–14). Recent data also suggest that GBS is a frequent cause of chorioamnionitis, endometritis, pneumonia, and urosepsis in adults with underlying medical conditions (i.e., diabetes mellitus or immunosuppression) (15–19). As these patterns of pathogenesis suggest, GBS is considered a saprophytic organism, i.e., invasive GBS disease is most commonly observed in weakened hosts.

Treatment of GBS disease relies primarily on penicillin and ampicillin, followed by first-generation cephalosporins and vancomycin (20). Alternative antibiotics, such as lincosamides, are used for patients with β-lactam allergies. Due to resistance evolution, macrolides, aminoglycosides, and tetracyclines are no longer clinically efficacious (21–24). While our group and others have observed that GBS is resistant to a wide range of antibiotics, GBS resistance remains poorly characterized and is a frontier of concern in the clinic.

In the early stages of this program, we hypothesized that human milk oligosaccharides (HMOs) possess antimicrobial and antivirulence properties (25). Indeed, we discovered that heterogeneous HMOs modulate growth and biofilm production for a number of bacterial pathogens (26, 27). We also determined the identities of several single-entity HMOs with potent antimicrobial activity against GBS (28–31). In addition to structure-activity relationship (SAR) studies, we found that HMOs potentiate the activity of select intracellular targeting antibiotics (32). This included three antibiotics to which GBS has evolved resistance, aminoglycosides, macrolides, and tetracyclines (33–42). At their 50% inhibitory concentration (IC_50_), HMO extracts reduced the MICs of certain intracellular targeting antibiotics up to 32-fold (Table 1). Interestingly, HMO treatment did not affect β-lactam or glycopeptide activity; antibiotics that interfere with cell wall synthesis. Based on this activity pattern, we hypothesized that HMOs function by increasing membrane permeability, which would be an unprecedented mode of action in GBS. This hypothesis was validated when HMOs were found to increase membrane permeability by ca. 30% using a LIVE/DEAD BacLight assay (32).

**TABLE 1:** Established patterns of HMO potentiation of antibiotic activity

Antibiotic in THB medium (strain)	MIC (μg/ml)		Fold reduction
	Overall	With 5.0 mg/ml HMOs	
Penicillin (CNCTC 10/84)	0.03	0.015	2
Vancomycin (CNCTC 10/84)	2	1	2
Clindamycin (GB2)	0.0312	0.0078	4
Gentamicin (GB590)	16	1	16
Erythromycin (GB590)	0.0312	0.001	32
Minocycline (CNCTC 10/84)	0.0625	0.0019	32

Based on their ability to increase cellular permeability, a second-generation combination study was initiated to further characterize HMO enhancement of intracellular targeting antibiotics in the GBS model. We took particular interest in trimethoprim (TMP), an antifolate used in the treatment of enteric, respiratory, skin, and urinary tract infections (43). Mechanistically, TMP is a bacteriostatic agent that inhibits dihydrofolate reductase (DHFR), an enzyme within the folate biosynthesis pathway (44). Importantly, interference with this pathway inhibits pyrimidine and purine biosynthesis, with downstream effects on bacterial DNA synthesis. Furthermore, a wide range of streptococcal strains, including GBS, are intrinsically resistant to TMP (45–51). Resistance is typically mediated by one of the following five mechanisms: (i) poor membrane permeability, (ii) an impervious DHFR, (iii) mutations in the inherent DHFR, (iv) upregulation of gene expression or gene duplication to increase DHFR production, and (v) horizontal transfer of *dfr* genes that encode resistant DHFRs (45). We hypothesized that if TMP has difficulty gaining penetrance into the GBS cell, HMOs could be used to sensitize GBS to TMP. Described herein are the results of testing this hypothesis using heterogeneous HMO extracts. To further evaluate the mechanism of HMO sensitization, ultrahigh-performance liquid chromatography–high-resolution tandem mass spectrometry analysis (UPLC-HRMS/MS) was used to characterize the immediate metabolic response of GBS to HMO-induced perturbations.

## RESULTS AND DISCUSSION

### HMOs demonstrate synergy with TMP against group B *Streptococcus*.

HMOs were isolated from donor breast milk and pooled to create two HMO cocktails; the first (HMO-1) used milk from 10 donors, while the second (HMO-2) used milk from 7 donors. Prior to potentiation studies, the MIC of the HMOs and TMP were determined separately in each strain of GBS grown in Todd-Hewitt broth (THB) using a broth microdilution assay (Table 2). HMOs were assayed against five strains of GBS of various serotypes to determine the strain specificity of antibiotic potentiation. The strains selected are all clinical isolates. CNCTC 10/84 is commercially available (52). Isolates GB00590, GB00002, GB00651, and GB00083 were recovered from colonized pregnant women (53, 54). GBS strains are divided into 10 serotypes (1a, 1b, and II to IX) based on a serological reaction against their capsular polysaccharides (55). GB2, GB590, and CNCTC 10/84 are serotypes Ia, III, and V, respectively. These three serotypes are the most common isolates associated with early-onset disease in the United States and together account for over 80% of all isolates (56). GB651 and GB83 are serotypes Ib and IV, respectively. Globally, the five strains represent 85% of all isolate serotypes (57).

**TABLE 2:** HMO potentiation of TMP

Strain in THB medium	MIC (μg/ml) for:			Fold reduction
	HMOs	TMP	TMP with 1.42 mg/ml HMO	
CNCTC 10/84	5.12^*a*^	>1,024	8^*a*^	≥256
GB2	2.56^*a*^	1,024	2^*a*^	512
GB590	5.12^*a*^	>1,024	32^*a*^	≥64
GB651	5.12^*b*^	512	32^*b*^	16
GB83	5.12^*b*^	>1,024	128^*b*^	≥16

a HMO-1.

b HMO-2.

HMOs were dosed at their 25% inhibitory concentrations (IC_25_s) in CNCTC 10/84 and GB2. The growth of the remaining GBS strains were so rapidly affected by HMO treatment that subsequent IC_50_ curve fitting yielded immeasurable confidence limits. For these strains, the IC_25_ from a similar strain having a superior fit dose-response curve was used (see Fig. S1A to E in the supplemental material). In each strain, the MIC of TMP was 512 μg/ml or higher (Table 2). In GB2, a 512-fold reduction in MIC was observed. A 256-fold reduction in MIC was observed in CNCTC 10/84. For the three remaining strains (GB651, GB83, and GB590), the fold reductions in MIC were 16, 16, and 64, respectively. The potentiation patterns described above are remarkable for several reasons. First, they represent the greatest magnitude of antibiotic enhancement that we have observed. Second, GBS is not susceptible to antifolate antibiotics, so the chemotherapeutic regime is effective at sensitizing GBS to TMP.

10.1128/mBio.00076-20.1 FIG S1 (A) IC_50_ curve of HMO cocktail for GB2 in THB. Bacterial growth (OD_600_) was recorded after 24 h of HMO treatment at increasing HMO concentrations. MIC, 2.56 mg/ml; IC_50_, 1.897 mg/ml; IC_25_, 1.43 mg/ml; Hill slope, −3.894. (B) IC_50_ curve of HMO cocktail for GB590 in THB. Bacterial growth (OD_600_) was recorded after 24 h of HMO treatment at increasing HMO concentrations. MIC = 5.125 mg/ml, IC_50_ ∼ 2.417 mg/ml, IC_25_ ∼2.30 mg/ml, hill slope = −22.37. (C) IC_50_ curve of HMO cocktail for GB CNCTC 10/84 in THB. Bacterial growth (OD_600_) was recorded after 24 h of HMO treatment at increasing HMO concentrations. MIC = 5.125 mg/ml, IC_50_ = 2.05 mg/ml, IC_25_ = 1.42 mg/ml, hill slope = −2.98. (D) IC_50_ curve of HMO cocktail for GB83 in THB. Bacterial growth (OD_600_) was recorded after 24 h of HMO treatment at increasing HMO concentrations. MIC = 5.125 mg/ml, IC_50_ = 3.616 mg/ml, IC_25_ = 3.51 mg/ml, hill slope = −39.06. (E) IC_50_ curve of HMO cocktail for GB651 in THB. Bacterial growth (OD600) was recorded after 24 h of HMO treatment at increasing HMO concentrations. MIC = 5.125 mg/ml, IC_50_ = 2.616 mg/ml, IC_25_ = 2.33 mg/ml, hill slope = −9.639. Download FIG S1, DOCX file, 0.1 MB. Copyright © 2020 Chambers et al. 2020 Chambers et al. This content is distributed under the terms of the Creative Commons Attribution 4.0 International license.

Next, checkerboard assays were conducted with GB2 and GB590, stains for which strong and weak potentiation of TMP were observed, respectively, to determine if the HMO-TMP combination was synergistic or additive in nature (Fig. S2A and B). Synergy is measured using the fractional inhibitory concentration (FIC) index value and is defined when the FIC is ≤0.5 for each combination of compounds. It was demonstrated that in GB590, synergy was achieved when dosing HMOs from 1.28 to 2.56 mg/ml in combination with TMP dosed at 8 to 128 μg/ml (ΣFIC values, 0.281 to 0.508). In GB2, the combination was synergistic with treatment of HMOs between 0.64 and 1.28 mg/ml in conjunction with TMP at 4 to 32 μg/ml (ΣFIC values, 0.281 to 0.508). These assays firmly demonstrate the HMO-TMP combination to be synergistic, and they characterize the dosing windows required to achieve this effect.

10.1128/mBio.00076-20.2 FIG S2 (A) Checkerboard assay in GB2 showing the synergistic combinations between TMP and the HMO cocktail (bolded). (B) Checkerboard assay in GB590 showing the synergistic combinations between TMP and the HMO cocktail (bold). The combination is considered synergistic when the ΣFIC is ≤0.5, additive or indifferent when the ΣFIC is >0.5 to <4, and antagonistic when the ΣFIC is ≥4. Download FIG S2, DOCX file, 0.1 MB. Copyright © 2020 Chambers et al. 2020 Chambers et al. This content is distributed under the terms of the Creative Commons Attribution 4.0 International license.

After evaluating the level of synergy in the cocktail, we conducted an experiment to validate whether growth inhibition was due to HMO enablement of cognate engagement of TMP with the folate pathway. By inhibiting folate biosynthesis, antifolates inhibit the *de novo* biosynthesis of thymidylate and purine nucleotides. However, in addition to *de novo* synthesis, cells can produce these nucleotides via salvage pathways that use free thymidine or purine bases as precursors for the corresponding nucleotides. Thus, we hypothesized that if HMOs facilitate TMP inhibition of the *de novo* synthesis pathway, the addition of the preformed nucleotide precursors thymidine or hypoxanthine would dampen the growth inhibitory capabilities of the HMO-TMP combination, as thymidine and hypoxanthine serve as precursors in the pyrimidine and purine salvage pathways, respectively (58).

In the experiment, we evaluated the MIC of the HMO-TMP cocktail in the presence of thymidine (Table 3). The experiment was conducted in strains GB2 and GB590. Against GB2, HMO supplementation decreased the MIC of TMP from 1,024 μg/ml to 2 μg/ml (512-fold reduction). Against GB590, the MIC of TMP was reduced from >1,024 μg/ml to 32 μg/ml (at least a 64-fold reduction) (Table 2). In the presence of added thymidine, the MICs of TMP in the HMO-TMP combination increased 8-fold to 16 μg/ml in GB2 and 4-fold to 128 μg/ml in GB590. These results support the hypothesis that supplemental thymidine mitigates the effects of the HMO-TMP combination and is able to partially salvage the folate biosynthetic pathway. Importantly, the MIC of the HMO cocktail individually did not change in the presence of added thymidine. This indicates that the folate pathway is not a target for HMOs. We therefore conclude that, in the presence of HMOs, TMP gains penetrance into the group B streptococcal cell and exhibits on-target inhibition of the folate cycle.

**TABLE 3:** HMO potentiation of TMP in the presence of thymidine

Strain in THB medium plus 20 μg/ml thymidine	MIC (μg/ml) for:		Fold reduction
	TMP	TMP with HMO-1 (dose [mg/ml])	
GB2	1,024	16 (1.43)	64
GB590	>1,024	128 (1.42)	8

The final assay in this study was a comparison of the HMO-TMP combination with the clinically useful TMP-sulfadiazine (SDZ) combination (Table S2). In both GB590 and GB2, the TMP-sulfadiazine combination was largely ineffective, with an MIC of ≥512 μg/ml, while the HMO-TMP combination sees the potentiation profile described above (Table 2). This result demonstrates that while antifolate-based antibiotic combination treatments remain largely ineffective against GBS, the HMO-TMP combination is operative. This insight offers new consideration for the use of existing combination therapies in patient care.

### Characterizing the HMO mode of action using untargeted metabolomics.

The mode of action of an antimicrobial agent cannot accurately be described in terms of a single static target; rather, the complete induced response must be evaluated. In theory, a single chemotherapeutic could have a wide range of direct and indirect targets, simultaneously interfering with multiple enzymes or pathways. Accordingly, the final stage of the study focused on utilizing global, untargeted metabolomic analysis to characterize the early response of GBS to HMO-mediated perturbations. The analysis described provides HMO-mediated perturbations. For this study, strain GB2 was used, as it was most susceptible to treatment with HMOs (MIC, 2.56 μg/ml). Two groups were analyzed and compared, with the first being an untreated GB2 control and the second being GB2 treated with HMOs dosed at 1 mg/ml. This concentration promoted cellular death (ca. 20 to 40%) compared to the untreated controls but also provided enough remaining cellular mass for analysis (minimum, 200 μg).

**FIG 1:**
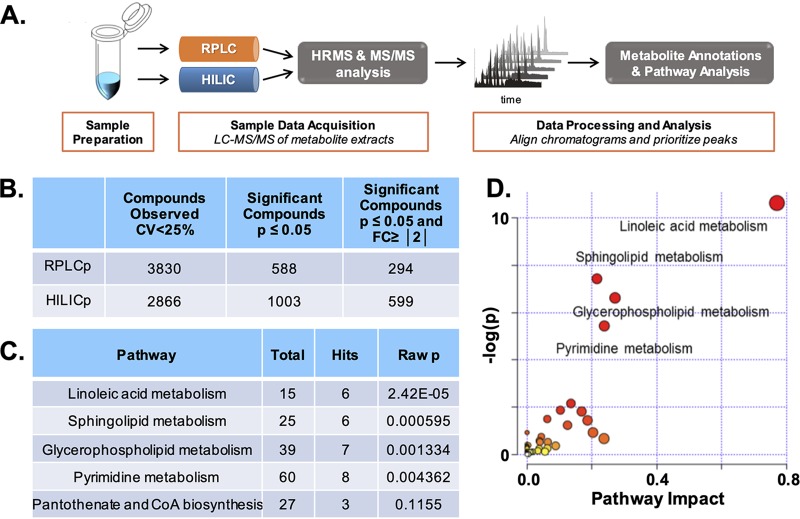
Workflow and pathway analysis using global, untargeted metabolomics data analysis. (A) Overview of global, untargeted metabolomic workflow. (B) Global output of identified metabolites from RPLC and HILIC methods and subsequent filtering for significance according to a *P* value of ≤0.05 and fold change of ≥|2|. (C) Table output of metabolic pathway enrichment analysis. The number of total metabolites in the pathway, the number of hits, and the *P* value were calculated using MetaboAnalyst 4.0. CoA, coenzyme A. (D) Metabolomic pathway analysis visualization. Shown is a graphical representation analysis using the statistically significant metabolite compounds (*P* ≤ 0.05; fold change, ≥|2|) annotated from RPLC and HILIC analyses. Matched pathways were arranged by *P* values (from pathway enrichment analysis) on the *y* axis, and pathway impact values (from pathway topology analysis) are shown on the *x* axis; node color is based on pathway *P* value, and node radius is determined based on pathway impact values; individual nodes represent individual pathways.

**FIG 2:**
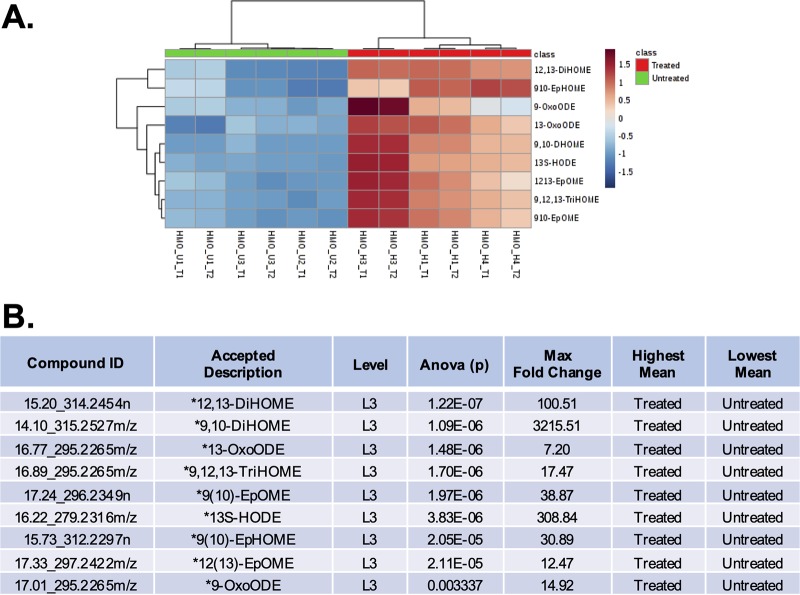
Linoleic acid-associated metabolite identification and statistical representation. (A) Heat map visualization of the significantly differently regulated linoleic acid metabolic pathway upon HMO treatment. Linoleic acid metabolism members shown here were detected by RPLC-positive LC-MS/MS analysis. Samples (columns) and metabolite compounds (rows) were processed using Euclidean average clustering via MetaboAnalyst 4.0. The heat map was generated for Pareto-scaled, log-transformed data, and colors are displayed by relative abundance, ranging from low (blue) to high (red), as shown in the legend. (B) Corresponding data table of linoleic acid metabolites, where the asterisk (*) denotes significance with a *P* value of ≤0.05 and fold change of ≥|2|. ODE, octadecadienoic acid; 13-HOTE, 13-hydroxyoctadeca-9,11,15-trienoic acid; 9,10-DiHOME, 9,10-dihydroxyoctadec-12-enoic acid; 9,12,13-TriHOME, 9,12,13-trihydroxyoctadecanoic acid; 13S-HODE, 13S-octadecadienoic acid; 9(10)-EpHOME, 9(10)-epoxyhydroxyoctadecanoic acid; ID, identifier.

**FIG 3:**
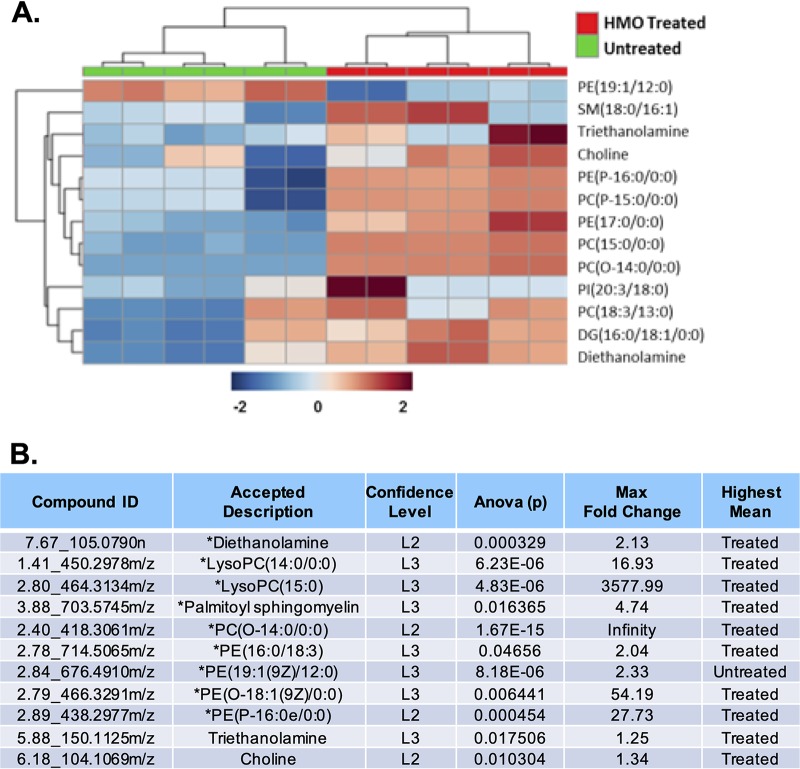
Glycerophospholipid-associated metabolite identification and statistical representation. (A) Heat map visualization of the significantly differently regulated glycerophospholipid metabolism pathway upon HMO treatment. Glycerophospholipid members shown here were detected by HILIC-positive LC-MS/MS analysis. Samples (columns) and metabolite compounds (rows) were processed using Euclidean average clustering via MetaboAnalyst 4.0. The heat map was generated for Pareto-scaled, log-transformed data, and colors are displayed by relative abundance, ranging from low (blue) to high (red), as shown in the legend. SM, sphingomyelin; PC, phosphocholine; PI, phosphoinositol; DG, diglyceride; LysoPC, lysophosphatidylcholine. (B) Corresponding data table of glycerophospholipid metabolites, where the asterisk (*) denotes significance with a *P* value of ≤0.05 and fold change of ≥|2|.

Our experimental design from sample collection through data analysis is depicted in Fig. 1A. The annotated and statistically significant metabolites observed in the experiment (Fig. 1B) were subjected to traditional pathway analysis (Fig. S3). The results showed the most statistically perturbed metabolic pathways to be linoleic acid metabolism, sphingolipid metabolism, glycerophospholipid metabolism, and pyrimidine metabolism (Fig. 1C and D). Characterized below are perturbations to both linoleic acid and glycerophospholipid metabolism (Fig. 2 and 3). We focus on these pathways, as each is critical to membrane formation and structural integrity, i.e., each pathway contributes to the synthesis of membrane-bound macromolecules and their corresponding precursors (59).

10.1128/mBio.00076-20.3 FIG S3 Global principal-component analysis (PCA) of cellular extracted metabolites for the two biological conditions is illustrating a distinct shift in metabolic profiles between groups. Presenting the abundance data in PC space allows us to separate the run samples based on overall variability and shows a clear separation of untreated samples versus HMO-treated samples across the first PC dimension (*x* axis). Download FIG S3, DOCX file, 0.1 MB. Copyright © 2020 Chambers et al. 2020 Chambers et al. This content is distributed under the terms of the Creative Commons Attribution 4.0 International license.

Based on statistical significance, linoleic acid metabolism is the metabolic pathway most impacted when GBS is exposed to HMOs (Fig. 2A and S4). Linoleic acid metabolites play a critical role in both cellular signaling and the stress response. Each is also critical to proper membrane construction (60, 61). All identified linoleic acid metabolites were accumulated in the HMO-treated population, with several metabolites having a >100-fold increase from the untreated controls (Fig. 2B). Two epoxyoctadecanoic acid metabolites were of particular interest, epoxyoctadecanoic acids (EpOMEs) and dihydroxyoctadecanoic acids (DiHOMEs). Accumulation of these metabolites is linked to changes in Na^+^ and K^+^ ion channels and, subsequently, cell membrane fluidity (62). In addition to the roles of EpOMEs and DiHOMEs in cell membrane construction, linoleic acid metabolites have been shown to have a critical role in cellular signaling and the stress response.

10.1128/mBio.00076-20.4 FIG S4 Linoleic acid metabolism supplementary images. (A) Heat map visualization of the differently regulated members within the linoleic acid metabolic pathway upon HMO treatment. Linoleic acid metabolism members shown here were detected by HILIC-positive LC-MS/MS analysis. (B) Red nodes reveal individual significant metabolites matched to the linoleic acid metabolic pathway. The KEGG C number is shown within a node. The individual significant metabolites (*P* < 0.05) identified at confidence level L3 are highlighted in red for the linoleic acid metabolism, generated by the MetaboAnalyst 4.0 software. Download FIG S4, DOCX file, 0.2 MB. Copyright © 2020 Chambers et al. 2020 Chambers et al. This content is distributed under the terms of the Creative Commons Attribution 4.0 International license.

Glycerophospholipid metabolism was also significantly impacted, and in general, we observed an accumulation of these metabolites compared to the control (Fig. 3A and S5). Glycerophospholipid metabolites were observed with significant fold changes compared to the control. For example, PE(17:0/0:0), PE(P-16:0/0:0), and PE(19:1/12:0), known degradation products of phosphatidylethanolamine (PE), one of three major components of the cellular membrane, were observed to have up to a 50-fold change increase compared to the untreated control (63). This observed accumulation of lipid metabolites indicates an increased rate of breakdown of critical cell membrane components when bacteria are dosed with HMOs. In fact, this type of metabolite accumulation has been previously observed in other model organisms when exposed to antibiotics (64–67).

10.1128/mBio.00076-20.5 FIG S5 Glycerophospholipid metabolism supplementary images. (A) Heat map visualization of the differently regulated members within the glycerophospholipid metabolic pathway upon HMO treatment. Glycerophospholipid metabolism members shown here were detected by RPLC-positive LC-MS/MS analysis. (B) Red nodes reveal individual significant metabolites matched to the glycerophospholipid metabolic pathway. The KEGG C number is shown within a node. The individual significant metabolites (*P* < 0.05) identified at confidence level L3 are highlighted in red for the glycerophospholipid metabolism, generated by the MetaboAnalyst 4.0 software. Download FIG S5, DOCX file, 0.4 MB. Copyright © 2020 Chambers et al. 2020 Chambers et al. This content is distributed under the terms of the Creative Commons Attribution 4.0 International license.

In addition to dysregulating pathways directly related to the production of membrane components, HMOs perturbed additional pathways related to essential cellular function. These include, for example, increased accumulation of purine and pyrimidine nucleotide precursors. An inability to synthesize nucleotides would lead to perturbations in DNA and RNA synthesis. HMO treatment also led to an accumulation of metabolites in the cysteine and methionine metabolic pathway. Cysteine biosynthesis is the primary pathway for incorporating sulfur into cellular components. In addition to serving as a precursor of methionine, cysteine is also the direct precursor to biotin, thiamine, and lipoic acid. Methionine is an essential amino acid in all organisms, as it is both proteinogenic and a component of the cofactor *S*-adenosyl methionine. Interestingly, sphingolipid metabolism was observed to be significantly perturbed upon HMO treatment. While GBS does not synthesize sphingolipids directly, it does rely on the host for access to these compounds for the biosynthesis of cell membrane components. This metabolic change could suggest that HMO treatment has an impact on host-microbe interactions (68, 69). Finally, several cell wall synthesis-associated metabolites were also identified as being accumulated in the treated sample, but a higher experimental mass range would be needed to identify a more significant amount of these precursors and better identify a global trend. The variety of metabolic pathways perturbed not only illuminates the synergistic nature of the TMP and HMO combination treatment but demonstrates that antimicrobial agents have broad effects on cellular biology (Fig. S6).

10.1128/mBio.00076-20.6 FIG S6 Heat map visualization of the differently regulated members within the cell wall synthesis-associated metabolites upon HMO treatment. Metabolites shown here were detected by HILIC-positive LC-MS/MS analysis. Download FIG S6, DOCX file, 0.1 MB. Copyright © 2020 Chambers et al. 2020 Chambers et al. This content is distributed under the terms of the Creative Commons Attribution 4.0 International license.

In summary, this study demonstrates that HMOs potentiate trimethoprim in group B *Streptococcus* bacteria, with a synergistic profile that spans the most prevalent serotypes worldwide. This potentiation profile makes antifolate antibiotics of potential use in an organism where they have been long considered resistant. Moreover, this combination could represent an alternative treatment for GBS-positive mothers with penicillin allergies given the high rates of resistance observed with alternative agents.

To characterize the mechanism of HMO-mediated antimicrobial activity, we have presented the first global, untargeted metabolomic analysis of HMO-mediated perturbations within any cell type and have shown significant impacts on cell membrane-affiliated macromolecules. While a number of high-throughput methods have been performed to elucidate an antibiotic’s mode of action (e.g., cytological profiling, genetic screens, or gene expression and proteomic profiling), direct experimental evidence that rapid metabolic changes are causal in facilitating the microbial response to antibiotics is lacking. In fact, little is described about the downstream phenotypic changes induced by antimicrobial agents. In the future, metabolomic experiments will be employed to better describe the phenotypic response of GBS to HMO-induced effects. We hypothesize that metabolomics will enable the characterization of the indirect connections critical to HMOs’ mechanism of action. Since metabolites present the final phenotypic manifestation of an organism and the final endpoint of biochemical reactions reflects the interplay between gene expression, protein function, and the environment, we argue that further metabolomics analyses are necessary to understand the HMO mode of action (70, 71).

## MATERIALS AND METHODS

### Antibiotics and additional chemicals.

Trimethoprim lactate 98% was purchased from Alfa Aesar. β-Galactosidase from Kluyveromyces lactis, at 2,600 units/g, was purchased from Sigma-Aldrich. Acetonitrile (ACN; catalog no. A955-1), methanol (MeOH; catalog no. A456-1), and water (catalog no. W6-1, liquid chromatography-mass spectrometry [LC-MS] grade; Optima) for the mass spectrometry analysis were obtained from Thermo Fisher Scientific.

### HMO isolation.

Human milk was obtained from 17 healthy, lactating women between 3 days and 3 months postpartum and stored between −80 and –20°C. Deidentified milk was provided by Jörn-Hendrik Weitkamp from the Vanderbilt Department of Pediatrics, under a collection protocol approved by the Vanderbilt University institutional review board (IRB no. 100897), or from Medolac. Milk samples were thawed and then centrifuged for 45 min. Following centrifugation, the resultant top lipid layer was removed. The proteins were then removed by diluting the remaining sample with roughly 1:1 (vol/vol) 180 or 200 proof ethanol, chilling the sample briefly, and centrifuging for 45 min, followed by removal of the resulting HMO-containing supernatant. Following concentration of the supernatant *in vacuo*, the HMO-containing extract was dissolved in 0.2 M phosphate buffer (pH 6.5) and heated to 37°C. β-Galactosidase from Kluyveromyces lactis was added, and the reaction mixture was stirred until lactose hydrolysis was complete. The reaction mixture was diluted with roughly 1:0.5 (vol/vol) 180 or 200 proof ethanol, chilled briefly, and then centrifuged for 30 min. The supernatant was removed and concentrated *in vacuo*, and the remaining salts, glucose, and galactose were separated from the oligosaccharides using size exclusion chromatography with P-2 gel (H_2_O eluent). The oligosaccharides were then dried by lyophilization. Correspondingly, HMO isolates from donors were combined and solubilized in water to reach a final concentration of 102.6 mg/ml.

### Bacterial strains and culture conditions.

The bacterial strains are shown in Table S1. All strains were grown on tryptic soy agar plates supplemented with 5% sheep blood (blood agar plates) at 37°C in ambient air overnight. All strains were subcultured from blood agar plates into 5 ml of Todd-Hewitt broth (THB) and incubated under shaking conditions at 180 rpm at 37°C overnight. Following overnight incubation, bacterial density was quantified through absorbance readings at 600 nm (OD_600_) using a Promega GloMax-Multi detection system plate reader. Bacterial numbers were determined using the predetermined coefficient of an OD_600_ of 1, equal to 10^9^ CFU/ml.

10.1128/mBio.00076-20.7 TABLE S1 Group B *Streptococcus* strains evaluated in the study. *ST, sequence type as defined by multilocus sequence typing (MLST). Download Table S1, DOCX file, 0.1 MB. Copyright © 2020 Chambers et al. 2020 Chambers et al. This content is distributed under the terms of the Creative Commons Attribution 4.0 International license.

10.1128/mBio.00076-20.8 TABLE S2 TMP-sulfadiazine antibiotic combination assay. *^a^*THB medium. *^b^*Shown in μg/ml. *^c^*1:4.85 ratio of TMP-sulfadiazine. Download Table S2, DOCX file, 0.1 MB. Copyright © 2020 Chambers et al. 2020 Chambers et al. This content is distributed under the terms of the Creative Commons Attribution 4.0 International license.

### Broth microdilution method for determination of MICs.

All strains were grown overnight as described above and used to inoculate fresh THB or THB plus 20 μg/ml thymidine to achieve 5 × 10^5^ CFU/ml. To 96-well tissue culture-treated, sterile polystyrene plates was added the inoculated medium in the presence of increasing concentrations of antibiotic or HMO cocktail to achieve a final volume of 100 μl per well. Bacteria grown in medium in the absence of any compounds served as the controls. The plates were incubated under static conditions at 37°C in ambient air for 24 h. Bacterial growth was quantified through absorbance readings (OD_600_). The MICs were assigned at the lowest concentration of compound at which no bacterial growth was observed.

### Broth microdilution method for antibiotic combination.

All strains were grown overnight as described above and the subcultures used to inoculate fresh THB or THB plus 20 μg/ml thymidine to achieve 5 × 10^5^ CFU/ml. Freshly inoculated medium was then supplemented with HMOs at their IC_25_. To 96-well tissue culture-treated, sterile polystyrene plates was added the inoculated medium supplemented with HMOs in the presence of increasing concentrations of antibiotic. Bacteria grown in medium in the absence of any compounds served as one control. Bacteria grown in medium supplemented with HMOs in the absence of any antibiotic served as a second control. MICs were determined as previously described.

### Synergy assay.

Group B *Streptococcus* strains (GB2 and GB590) were grown overnight as described above and used to inoculate fresh THB to achieve 5 × 10^5^ CFU/ml. One hundred microliters per well of inoculated medium was added to 96-well tissue culture-treated, sterile polystyrene plates. Trimethoprim was 2-fold serially diluted descending down the plate to achieve a final volume of 100 μl per well. The final row was left without any trimethoprim. The HMO cocktail was 2-fold serially diluted going from right to left across the plate. The far-left column was left without any HMO cocktail. Bacteria grown in medium in the absence of either compound served as the controls. The plates were incubated under static conditions at 37°C in ambient air for 24 h. Bacterial growth was quantified through absorbance readings (OD_600_). The MICs were assigned at the lowest concentration of compound at which no bacterial growth was observed. The fractional inhibitory concentration (FIC) index was used to evaluate synergy. The calculation of the FIC index is as follows: ΣFIC = FIC A + FIC B = (MIC of drug A in the combination/MIC of drug A alone) + (MIC of drug B in the combination/MIC of drug B alone), where A is trimethoprim and B is the HMO cocktail. The combination is considered synergistic when the ΣFIC is ≤0.5, additive or indifferent when the ΣFIC is >0.5 to <4, and antagonistic when the ΣFIC is ≥4.

### Statistical analysis.

The data for the HMO antimicrobial and combination assays represent 3 biological replicates, each with 3 technical replicates. The data for the synergy assays represent 3 biological replicates. Data are expressed as the mean biomass ± standard error of the mean (SEM). Statistical analyses were performed in the GraphPad Prism software v. 7.0c. Statistical significance was determined using one-way analysis of variance (ANOVA) with *post hoc* Dunnett’s multiple-comparison test comparing growth in the presence of ca. 5 mg/ml HMOs to growth in medium alone. HMO IC_50_ curves were generated in the GraphPad Prism software v. 7.0c using an inhibition dose-response nonlinear regression curve fit for log(inhibitor) versus normalized response with a variable slope.

### Sample preparation for metabolomic analysis.

Group B *Streptococcus* strain GB2 was grown overnight as described above and used to inoculate 10 ml of fresh THB medium to achieve 5 × 10^5^ CFU/ml. Untreated GB2 in 10 ml of medium served as a control, while other GB2 cultures were treated with HMOs at 1.00 mg/ml. After 24 h, the samples were centrifuged at 1,500 rpm for 20 min to generate a bacterial pellet. The medium was removed and the pellet washed with 200 μl of 50 mM ammonium formate buffer. The pellet was then resuspended in 200 μl of 50 mM ammonium formate buffer and transferred to a sterile Eppendorf tube. This was then centrifuged at 1,500 rpm for 10 min to generate a bacterial pellet. The buffer was removed and the pellet flash frozen in liquid N_2_ and stored until use.

The bacterial cell pellets were lysed using 400 μl ice-cold lysis buffer (1:1:2, AcCN:MeOH:ammonium bicarbonate 0.1 M [pH 8.0], LC-MS grade) and vortexed. Individual samples were sonicated using a probe tip sonicator, with 10 pulses at 30% power and cooling down in ice between samples. A bicinchoninic acid (BCA) protein assay was used to determine the protein concentration for each individual sample and adjusted to a total amount of protein of 200 μg in 200 μl of lysis buffer. Isotopically labeled standard molecules phenylalanine-D8 (CDN Isotopes, Quebec, CA) and biotin-D2 (CIL, MA, USA) were added to each sample to assess sample preparation reproducibility. Metabolites were extracted from untreated control and HMO-treated cultures using protein precipitation by the addition of 800 μl of ice-cold methanol (4× by volume) and incubated overnight at –80°C. Following incubation, samples were centrifuged at 10,000 rpm for 10 min to eliminate precipitated proteins, and the metabolite-containing supernatant was dried *in vacuo* and stored at –80°C until further UPLC-HRMS/MS analysis.

### Global untargeted metabolomic analyses.

Metabolite extracts were analyzed using reverse-phase liquid chromatography (RPLC) and hydrophilic interaction liquid chromatography (HILIC), followed by subsequent mass spectrometry analysis using a high-resolution Q-Exactive high-fidelity (HF) hybrid quadrupole-Orbitrap mass spectrometer (Thermo Fisher Scientific, Bremen, Germany) equipped with a Vanquish ultrahigh-performance liquid chromatography (UHPLC) binary system and autosampler (Thermo Fisher Scientific, Bremen, Germany). A quality control sample was prepared by pooling equal volumes of each sample. Isotopically labeled standards tryptophan-D3, carnitine-D9 (CDN Isotopes, Quebec, CA), valine-D8, and inosine-4N15 (CIL, MA, USA) were added to each sample to assess MS instrument reproducibility.

Metabolite extracts (10-μl injection volume) were separated on a SeQuant ZIC-HILIC 3.5-μm, 2.1-mm by 100-mm column (Millipore Corporation, Darmstadt, Germany) held at 40°C for the HILIC analysis. Liquid chromatography was performed at 200 μl/min using solvent A (5 mM ammonium formate in 90% water, 10% acetonitrile) and solvent B (5 mM ammonium formate in 90% acetonitrile, 10% water) with the following gradient: 95% B for 2 min, 95 to 40% B over 16 min, 40% B held for 2 min, and 40 to 95% B over 15 min, and 95% B held for 10 min (gradient length, 45 min). For the RPLC analysis, metabolite extracts (10 μl injection volume) were separated on a Hypersil Gold, 1.9 μm, 2.1-mm by 100-mm column (Thermo Fisher) held at 40°C. Liquid chromatography was performed at 250 μl/min using solvent A (0.1% formic acid [FA] in water) and solvent B (0.1% FA in acetonitrile [ACN]) with the following gradient: 5% B for 1 min, 5 to 50% B over 9 min, 50 to 70% B over 5 min, 70 to 95% B over 5 min, 95% B held for 2 min, 95 to 5% B over 3 min, and 5% B held for 5 min (gradient length, 30 min).

MS analyses were acquired over a mass range of *m/z* 70 to 1,050 using electrospray ionization positive mode. MS scans were analyzed at a resolution of 120,000, with a scan rate of 3.5 Hz. The automatic gain control (AGC) target was set to 1 × 10^6^ ions, and the maximum injection time (IT) was at 100 ms. Source ionization parameters were optimized, and these include spray voltage, 3.0 kV; transfer temperature, 280°C; S-lens level, 40; heater temperature, 325°C; sheath gas, 40; aux gas, 10; and sweep gas flow, 1. Tandem spectra were acquired using a data-dependent acquisition (DDA) in which one MS scan is followed by 2, 4, or 6 tandem MS (MS/MS) scans. MS/MS scans are acquired using an isolation width of *m/z* 1.3, stepped normalized collision energy (NCE) of 20 and 40, and a dynamic exclusion for 6 s. MS/MS spectra were collected at a resolution of 15,000, with an AGC target set at 2 × 10^5^ ions and maximum IT of 100 ms. Instrument performance and reproducibility in the run sequence were assessed by monitoring the retention times and peak areas for the heavy labeled standards added to the individual samples prior to and after metabolite extraction to assess sample processing steps and instrument variability (Table S3).

10.1128/mBio.00076-20.9 TABLE S3 Quality metrics obtained for the heavy labeled standard molecules used for this study to assess the metabolite extraction, instrument performance, and injection volume reproducibility. Download Table S3, DOCX file, 1.1 MB. Copyright © 2020 Chambers et al. 2020 Chambers et al. This content is distributed under the terms of the Creative Commons Attribution 4.0 International license.

### Metabolomics data processing.

UPLC-HRMS/MS raw data were imported, processed, normalized, and reviewed using Progenesis QI v.2.1 (Nonlinear Dynamics, Newcastle, UK). All MS and MS/MS sample runs were aligned against a quality control (pooled) reference run, and peak picking was performed on individual aligned runs to create an aggregate data set. Following peak picking, unique spectral features (retention time and *m/z* pairs) were grouped based on adducts and isotopes, and individual features or metabolites were normalized to all features. Compounds with <25% coefficient of variance (CV) were retained for further analysis. *P* values were calculated by Progenesis QI using variance-stabilized measurements achieved through log normalization, and metabolites with a *P* value of <0.05 calculated by one-way analysis of variance (ANOVA) and with a fold change (FC) of >|2| were considered significant.

Tentative and putative identifications were performed within Progenesis QI using accurate mass measurements (<5 ppm error), isotope distribution similarity, and fragmentation spectrum matching based on database searches against the Human Metabolome Database (HMDB), METLIN and National Institute of Standards and Technology (NIST) databases, and an in-house database (72–76). Annotations from both RPLC and HILIC analyses were performed for all significant compounds (*P* < 0.05, FC > |2|). Annotations were further analyzed using pathway overrepresentation analysis using MetaboAnalyst 4.0 (77, 78). The level system for metabolite identification confidence was used. The level 3 (L3) of confidence for the metabolite identifications was assigned for those molecules that showed minimal experimental evidence compared to level 2 (L2) but do prioritize a top candidate. These are accepted by the metabolomics community and represent families of molecules that cannot be distinguished by the data acquired, predominantly because there are too many isomers as possible candidate metabolites, but the family trends can be informative as well.
