# Supplementary figures and images for: A Solution to Antifolate Resistance in Group B Streptococcus: Untargeted Metabolomics Identifies Human Milk Oligosaccharide-Induced Perturbations That Result in Potentiation of Trimethoprim
Source: mBio. 2020 Mar 17;11(2):e00076-20. doi: 10.1128/mBio.00076-20 (PMC7078465; doi:10.1128/mBio.00076-20)

**HMO Cocktail IC50 Curves**

B.

D.
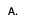
.

A.

C.

E.

Supplement: FIG S1 [file mBio.00076-20-sf001.docx]

**
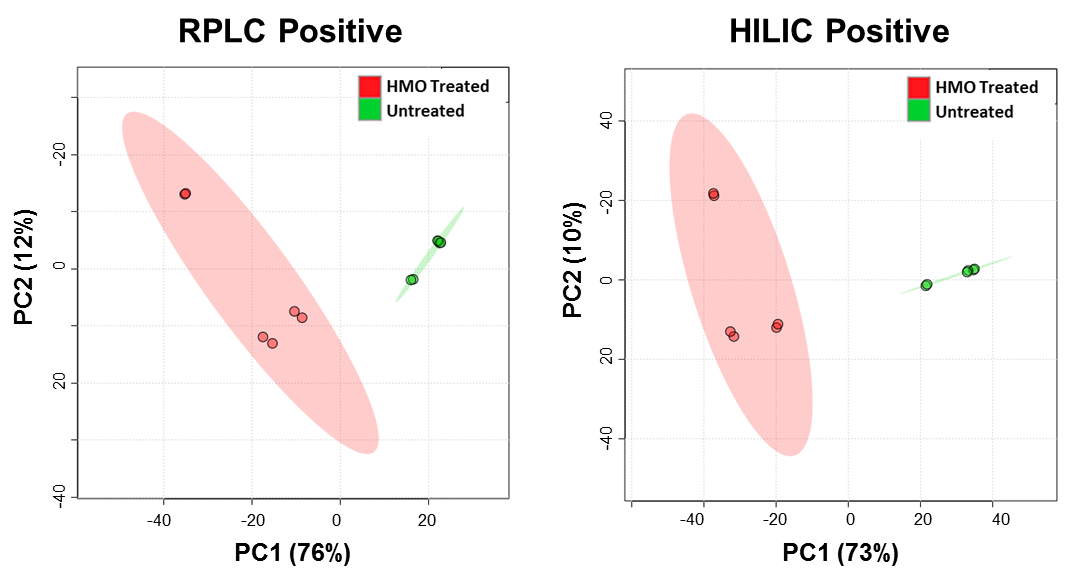
**

Supplement: FIG S3 [file mBio.00076-20-sf003.docx]

**
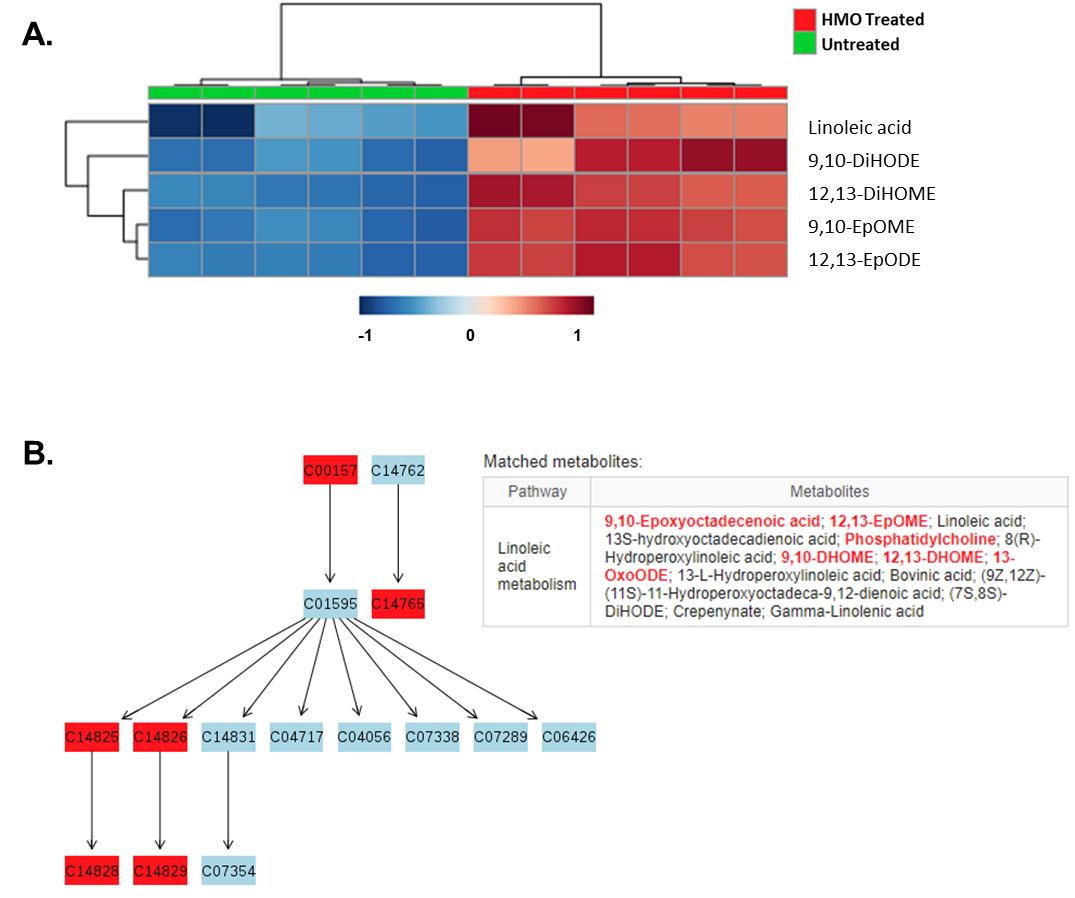
**

Supplement: FIG S4 [file mBio.00076-20-sf004.docx]

**
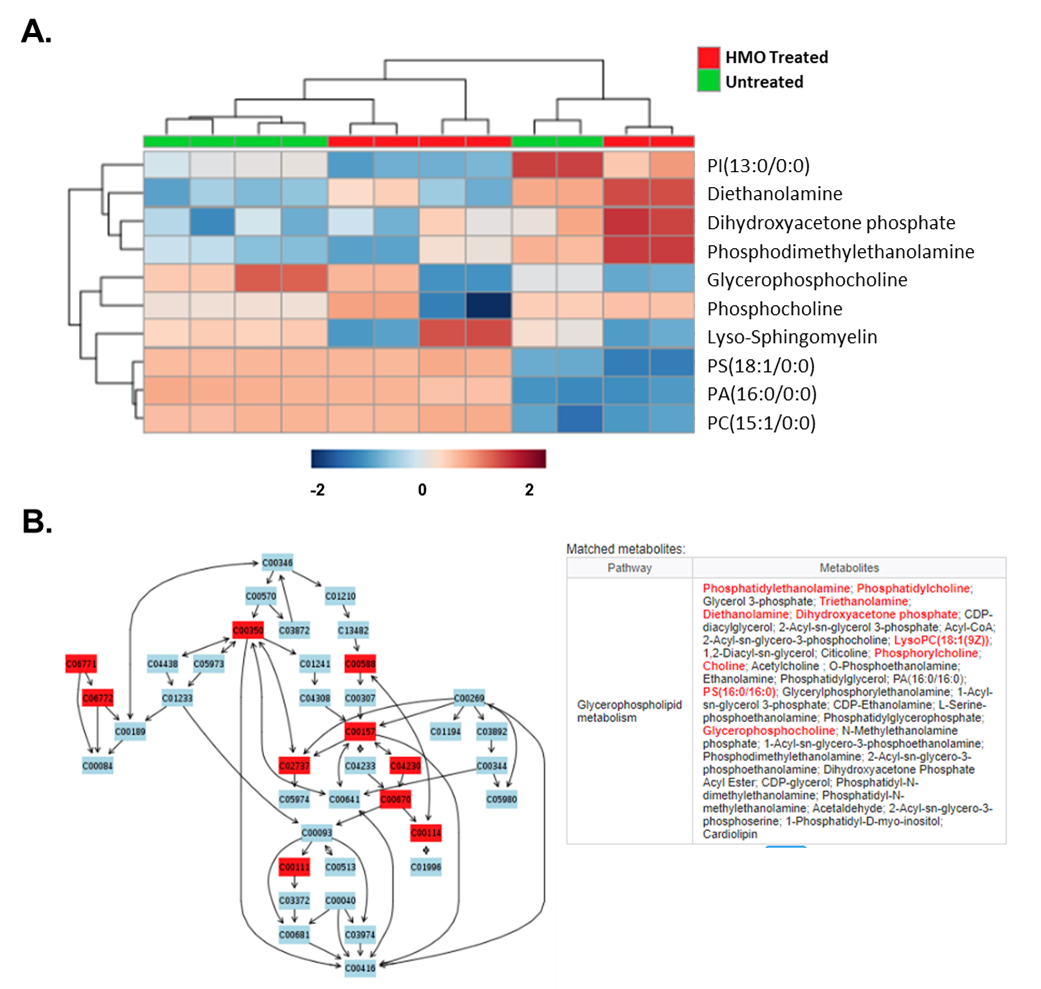
**

Supplement: FIG S5 [file mBio.00076-20-sf005.docx]

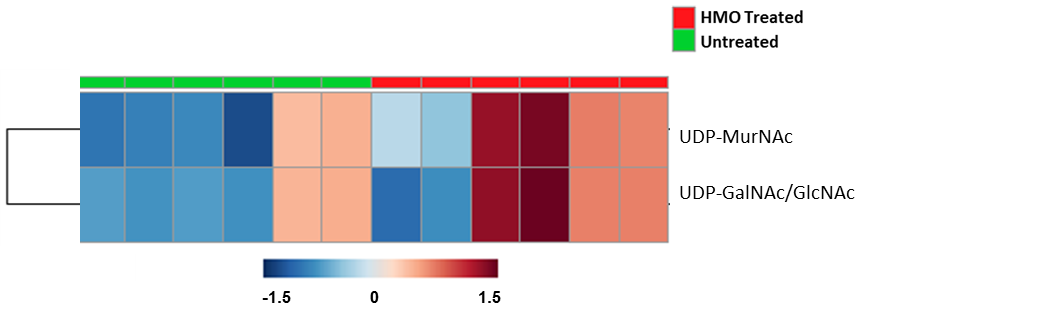

Supplement: FIG S6 [file mBio.00076-20-sf006.docx]

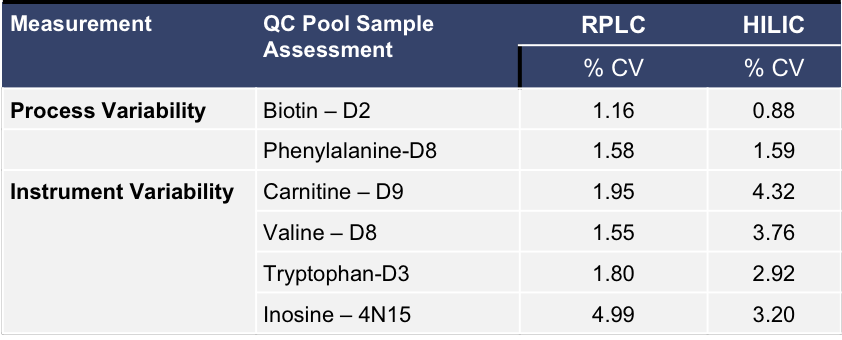

Supplement: TABLE S3 [file mBio.00076-20-st003.docx]
