# Supplementary material for: A Solution to Antifolate Resistance in Group B Streptococcus: Untargeted Metabolomics Identifies Human Milk Oligosaccharide-Induced Perturbations That Result in Potentiation of Trimethoprim
Source: mBio. 2020 Mar 17;11(2):e00076-20. doi: 10.1128/mBio.00076-20 (PMC7078465; doi:10.1128/mBio.00076-20)
Supplement: FIG S2 [file mBio.00076-20-sf002.docx]

**Checkerboard Assays**

A.

B.
