# Supplementary material for: A Solution to Antifolate Resistance in Group B Streptococcus: Untargeted Metabolomics Identifies Human Milk Oligosaccharide-Induced Perturbations That Result in Potentiation of Trimethoprim
Source: mBio. 2020 Mar 17;11(2):e00076-20. doi: 10.1128/mBio.00076-20 (PMC7078465; doi:10.1128/mBio.00076-20)
Supplement: TABLE S1 [file mBio.00076-20-st001.docx]

| **Strain** | **Serotype** | **ST *** | **Genome accession number** |
| --- | --- | --- | --- |
| GB00590 | III | 19 | NZ_LGAI01000000 |
| GB00002 | Ia | 23 | SAMN00991164 |
| CNCTC 10/84 | V | 26 | CP006910 |
| GB00083 | VI | 1 | SAMN00991156 |
| GB00651 | Ib | 8 | SAMN00991170 |
