# Supplementary material for: A Solution to Antifolate Resistance in Group B Streptococcus: Untargeted Metabolomics Identifies Human Milk Oligosaccharide-Induced Perturbations That Result in Potentiation of Trimethoprim
Source: mBio. 2020 Mar 17;11(2):e00076-20. doi: 10.1128/mBio.00076-20 (PMC7078465; doi:10.1128/mBio.00076-20)
Supplement: TABLE S2 [file mBio.00076-20-st002.docx]

| **Strain^a^** | **MIC of**  **TMP^b^** | **MIC of Sulfadiazine^b^** | **MIC of**  **TMP/Sulfadiazine Combination^b,c^** | **Fold Change** |
| --- | --- | --- | --- | --- |
| GB2 | 1024 | >64 | >512 | 0 |
| GB590 | >1024 | >64 | >512 | 0 |
